# Enhancing pediatric asthma management in underdeveloped regions through ChatGPT training for doctors: a randomized controlled trial

**DOI:** 10.3389/fped.2025.1519751

**Published:** 2025-07-03

**Authors:** Liwen Zhang, Guijun Yang, Jiajun Yuan, Shuhua Yuan, Jing Zhang, Jiande Chen, Mingyu Tang, Yunqin Zhang, Jilei Lin, Liebin Zhao, Yong Yin

**Affiliations:** ^1^ Department of Respiratory Medicine, Shanghai Children’s Medical Center, Shanghai Jiao Tong University School of Medicine, Shanghai, China; ^2^ Medical Department of Shanghai Children’s Medical Center, Shanghai Jiao Tong University School of Medicine, Shanghai, China; ^3^ Department of Respiratory Medicine, Linyi Maternal and Child Healthcare Hospital, Linyi Branch of Shanghai Children’s Medical Center, Linyi City, Shandong, China; ^4^ Pediatric AI Clinical Application and Research Center, Shanghai Children’s Medical Center, Shanghai, China; ^5^ Shanghai Engineering Research Center of Intelligence Pediatrics (SERCIP), Shanghai, China

**Keywords:** ChatGPT, pediatric, asthma management, large language models, randomized controlled trial

## Abstract

**Background:**

Childhood asthma represents a significant challenge globally, especially in underdeveloped regions. Recent advancements in Large Language Models (LLMs), such as ChatGPT, offer promising improvements in medical service quality.

**Methods:**

This randomized controlled trial assessed the effectiveness of ChatGPT in enhancing physicians' childhood asthma management skills. A total of 192 doctors from varied healthcare environments in China were divided into a control group, receiving traditional medical literature training, and an intervention group, trained in utilizing ChatGPT. Assessments conducted before and after training, and a 2-week follow-up, measured the training's impact.

**Results:**

The intervention group showed significant improvement, with scores of test questions increasing by approximately 20 out of 100 (improving to 72 ± 8 from a baseline, vs. the control group's increase to 50 ± 9). Post-training, ChatGPT's regular usage among the intervention group jumped from 6.3% to 62%, markedly above the control group's 4.3%. Moreover, physicians in the intervention group reported higher levels of familiarity, effectiveness, satisfaction, and intention for future use of ChatGPT.

**Conclusion:**

ChatGPT training significantly improves childhood asthma management among physicians in underdeveloped regions. This underscores the utility of LLMs like ChatGPT as effective educational tools in medical training, highlighting the need for further research into their integration and patient outcome impacts.

## 1 Introduction

Childhood asthma, a prevalent chronic respiratory condition among children, is traditionally characterized as an inflammation of the airways, manifested by symptoms such as coughing and wheezing. Furthermore, asthma is a multifaceted disease influenced by genetic predisposition, age, symptom severity, risk level, and comorbidities, posing significant risks to the affected children's growth and overall health. Initial studies conducted by the Global Asthma Network (GAN) reveal prevalence rates of asthma, rhinoconjunctivitis, and eczema in children at 11.0%, 13.3%, and 6.4% respectively (1). The hospitalizations and re-admissions for children with asthma amplify the economic strain on their families, profoundly affecting the children, their parents, and broader society (2–4). Severe childhood asthma represents an estimated 5%–10% of all asthma cases (5). Yet, the impact on these children and the healthcare system is substantial, with their care accounting for more than half of all asthma-related healthcare costs, largely due to increased resource use (6).

While wheezing, a primary indicator of childhood asthma, is more prevalent in developed countries, severe symptoms are notably more common in less developed regions (7). This trend may be attributed to underdiagnosis, inadequate disease management, recurrent infections, and prevalent indoor and outdoor air pollution in developing economies (8). China, embodying these challenges as a developing nation, confronts similar hurdles in managing childhood asthma. In many rural areas and small towns of developing countries, there is a notable shortage of trained pediatricians and pediatric pulmonologists. Consequently, children with asthma frequently receive care from general practitioners and adult pulmonologists. This lack of pediatric asthma awareness often leads to acute asthma attacks in young children being misdiagnosed as pneumonia. Consequently, young children frequently receive symptomatic treatment for acute wheezing without an asthma diagnosis (8). Reports indicate that in Beijing's rural areas, the asthma diagnosis rate stands at merely 48.9%, substantially lower than the 73.9% in urban locales, with only 35.6% of rural asthmatic children receiving regular inhaled corticosteroid treatment (9). There is an urgent need to enhance the medical management skills of doctors in economically underdeveloped areas for childhood asthma.

Large Language Models (LLMs), such as ChatGPT, leveraging advanced artificial intelligence for natural language processing and generation, demonstrate significant potential in medical applications (10–12). By sifting through extensive medical texts, including recent studies, clinical trial results, and patient histories, these models provide profound insights, aiding physicians in rendering more precise diagnoses and treatment choices (13, 14). Research underscores the promising utility of LLMs in managing conditions like prostate cancer, diabetes, and mental health disorders (15–17). The wealth of electronic medical records and test results from long-term consultations for childhood asthma showcases LLMs' capacity to synthesize and scrutinize complex medical data, highlighting their benefits in enhancing diagnostic accuracy, personalizing treatment plans, and boosting medical service efficiency. Despite LLMs' considerable benefits in healthcare, their adoption is challenged by issues such as data privacy and security, algorithmic bias, and the need for greater transparency and interpretability (14).

This study delves into the potential of LLMs to bolster physicians’ ability to manage childhood asthma, assessing the improvement in asthma competency scores between experimental and control groups.

## 2 Methods

### 2.1 Research design

This study recruited doctors from different economic regions and randomly assigned them into control and intervention groups. Firstly, we required all participants to complete the given tasks without any assistance (first round of testing). Subsequently, the intervention group received a 5-minute training on using ChatGPT, while the control group received a 5-minute training on medical literature search. Following the training, the next round of testing began, where the intervention group was asked to answer the previous tasks using ChatGPT 4.0 and the Pediatric Asthma Diagnosis and Treatment Guideline (18), while the control group used traditional internet search methods and the same guidelines (second round of testing). All participants were instructed to refer to the 2016 Chinese Pediatric Asthma Diagnosis and Treatment Guideline during testing. Although ChatGPT provided assistance in the intervention group, participants were asked to align their responses with this national guideline to ensure consistency across both groups.

To evaluate the long-term effectiveness of ChatGPT training, we conducted a survey via online questionnaire 2 weeks later to investigate the understanding and utilization of ChatGPT in both groups.

### 2.2 Participant recruitment

This study employed online recruitment from January 1, 2024, to January 14, 2024, targeting 64, 6, and 64 doctors respectively from tertiary hospitals in central and western regions, tertiary hospitals in the eastern region, and non-tertiary hospitals in the eastern region (19), representing doctors from economically underdeveloped regions, economically developed regions, and the edge of economically developed regions in China. Only participants meeting the following criteria were recruited: (1) holding a Chinese medical practitioner license; (2) seeing over 50 pediatric patients per week; (3) seeing over 5 asthmatic children per week; (4) proficient in using electronic devices such as computers and smartphones. Proficiency was assessed through self-report during recruitment, requiring participants to routinely use computers or smartphones in their clinical practice or medical learning. While we did not stratify outcomes by age or prior exposure to technology, this inclusion criterion helped ensure a basic level of digital literacy across participants. We also collected baseline information on each participant's duration of medical practice, which was summarized in Table 1 and included as a candidate variable in our regression analysis.

**Table 1:** The baseline of participants.

Characteristic	Overall	Eastern tertiary hospitals	Eastern non-tertiary hospitals	Central and western hospitals	*p*-value^b^
	*N* = 186	*N* = 61	*N* = 62	*N* = 63^a^	
Sex					0.2
Male	43 (23%)	14 (23%)	11 (18%)	18 (29%)	
Female	143 (77%)	47 (77%)	51 (82%)	45 (71%)	
Age (years)	42 (7)	41 (7)	43 (8)	42 (7)	0.5
Type of practice hospital					<0.001
Non-pediatric specialty	151 (81%)	50 (81%)	58 (94%)	43 (68%)	
Pediatric specialty	35 (18%)	11 (19%)	4 (6.5%)	20 (32%)	
Professional title					0.2
Associate chief physician	68 (35.5%)	28 (46%)	19 (31%)	21 (33%)	
Chief physician	17 (9%)	4 (6.6%)	4 (6.5%)	9 (14%)	
Attending physician	89 (48%)	24 (39%)	34 (55%)	31 (49%)	
Resident physician	12 (7.5%)	5 (8.4%)	5 (8.1%)	2 (3.2%)	
Practice Department					<0.001
Pediatric pulmonology	12 (6.5%)	7 (11%)	0 (0%)	5 (7.9%)	
Other pediatric internal medicine	148 (80%)	52 (85%)	41 (66%)	55 (87%)	
General practice	17 (9.1%)	0 (0%)	16 (26%)	1 (1.6%)	
Other departments	9 (4.4%)	2 (4%)	5 (8.1%)	2 (3.2%)	
Duration of medical practice (years)	17 (8)	16 (8)	18 (9)	17 (7)	0.3
Weekly asthma clinic sessions (half-day)	1.96 (1.19)	2.11 (1.07)	1.66 (1.09)	2.05 (1.40)	0.009
Number of asthma patients seen weekly	17 (27)	22 (30)	9 (14)	18 (31)	<0.001
Weekly pulmonary function tests	20 (177)	8 (17)	3 (6)	51 (319)	<0.001
Long-term follow-up numbers for asthma patients	20 (27)	26 (29)	15 (25)	18 (26)	0.012
Usage of ChatGPT					0.5
Never	173 (93%)	57 (93%)	56 (90%)	60 (95%)	
Previously	13 (7%)	4 (7%)	6 (9.7%)	3 (4.8%)	
Group					0.5
Control	92 (49%)	28 (46%)	34 (55%)	30 (48%)	
Treatment	94 (51%)	33 (54%)	28 (45%)	33 (52%)	
First Test Score	50 (11)	54 (10)	47 (12)	50 (9)	<0.001

^a^ *n* (%); mean (SD).

^b^ Pearson's Chi-squared test; Kruskal–Wallis rank sum test; Fisher's exact test, ANOVA.

To motivate participants to do their best, we informed them that they would receive generous cash rewards upon completing both rounds of testing, with additional substantial rewards based on performance rankings. We provided each participant with a cash reward of 100 RMB (exceeding their hourly wage), and an additional cash reward of 1,000 RMB for the top three performers. To minimize attrition, after completing the tests, participants were informed of a follow-up visit 2 weeks later, with continued provision of generous cash rewards.

### 2.3 Participant randomization

After completing participant recruitment, we randomly assigned participants in a 1:1 ratio to the control and intervention groups. To maintain blinding, we opted for simple randomization instead of stratified randomization for group assignment. Random group allocation was conducted using random numbers generated by computer programs.

### 2.4 Blinding

During participant recruitment, participants were only informed about the need to answer a set of asthma-related test questions without disclosing their group assignment or the differences between groups. Participants were informed of their group assignment after the first round of testing. Testing for both groups was conducted simultaneously to ensure no information leakage between the groups. Blinding was not relevant for the researchers.

### 2.5 Test questions and outcome measures

For citation purposes, we selected test questions mentioned in “Asthma guidelines: an assessment of physician understanding and practice” (20). For subsequent analysis, based on the aforementioned study, we analyzed participants' scores in terms of Assessment, Diagnosis, Pathophysiology, Pharmacology, Prevention, and Therapy. We chose test scores as objective indicators, while also requesting participants to rate the difficulty of the questions, satisfaction with their answers, the extent of assistance from auxiliary tools, and the usefulness of guidelines (all rated on a scale of 1–10). During the questionnaire follow-up, we surveyed whether participants continued using ChatGPT after the training, duration of usage, and various other indicators to evaluate the long-term effectiveness of the intervention.

### 2.6 Ethical approval and informed consent

We obtained ethical approval from the Ethics Committee of Shanghai Children's Medical Center, affiliated with Shanghai Jiao Tong University (Protocol Number: SCMCIRB-K2024047-1). A research informed consent form was placed on the first page of the questionnaire, and informed consent was obtained from all participants before testing.

### 2.7 Statistical analysis

All analyses were conducted using R version 4.3.2. Depending on variable types, we employed Pearson's Chi-squared test, Wilcoxon rank sum test, and ANOVA to assess the significance of baseline differences. Multiple linear regression was utilized to identify factors associated with scores from the first round of testing, and LASSO was employed for feature selection, including Sex, Age, Type of practice hospital, Professional title, Practice Department, Duration of Medical Practice, Weekly Asthma Clinic Sessions, Number of Asthma Patients Seen Weekly, Weekly Pulmonary Function Tests, Long-term Follow-up Numbers for Asthma Patients, Usage of ChatGPT and group. Paired *t*-tests were used to examine differences between the first and second rounds of testing. When analyzing follow-up data, we employed ANOVA to analyze differences between different groups.

## 3 Results

### 3.1 Baseline

A total of 192 doctors were recruited for this study. After excluding 6 participants who did not complete both rounds of testing, a total of 186 individuals were included. Among them, 92 were in the control group, and 94 were in the intervention group. Table 1 provides detailed information on the participants' demographics. Participants from non-tertiary hospitals in the eastern region were more likely to practice in non-pediatric specialty hospitals, with a higher proportion being general practitioners. Additionally, participants from non-tertiary hospitals in the eastern region had significantly lower numbers of Weekly Asthma Clinic Sessions, Number of Asthma Patients Seen Weekly, Weekly Pulmonary Function Tests, and Long-term Follow-up Numbers for Asthma Patients compared to the other two groups. Regarding the scores in the first round of testing, doctors from tertiary hospitals in the eastern region achieved the highest scores, followed by those from hospitals in central and western regions, while doctors from non-tertiary hospitals in the eastern region scored the lowest. No other significant statistical differences were observed in other characteristics.

### 3.2 Factors associated with scores in the first round

To verify the negative correlation between geographical regions and asthma management capabilities and to explore confounding factors, we employed Lasso for feature selection and multiple linear regression for modeling (Supplementary Figure S1). Ultimately, three variables were included in the model. Doctors from hospitals in central and western regions scored lower than those from tertiary hospitals in the eastern region (*β* = −3.57, 95% CI: −6.77 to −0.36). General practitioners (*β* = −15.77, 95% CI: −23.11 to −8.44) and doctors from other departments (*β* = −14.73, 95% CI: −22.78 to −6.68) scored lower than those from pediatric respiratory departments. For each unit increase in weekly asthma clinic visits, the score increased by 1.87 points. Table 2 displays these results.

**Table 2:** Multiple linear regression of scores in the first round of testing, including All features selected by LASSO.

Characters	*β* (95%CI)	*p*
Hospitals		
Eastern tertiary hospitals	/	/
Eastern non-tertiary hospitals	−2.13 (−5.73, 1.47)	0.245
Central and western hospitals	−3.57 (−6.77, −0.36)	0.029
Practice department		
Pediatric pulmonology	/	/
Other pediatric internal medicine	−4.17 (−9.12, 0.78)	0.099
General practice	−15.77 (−23.11, −8.44)	<0.001
Other departments	−14.73 (−22.78, −6.68)	<0.001
Weekly asthma clinic sessions (half-day)	1.87 (0.72, 3.01)	0.002

### 3.3 Detailed comparison of the first round of testing

**Figure 1:**
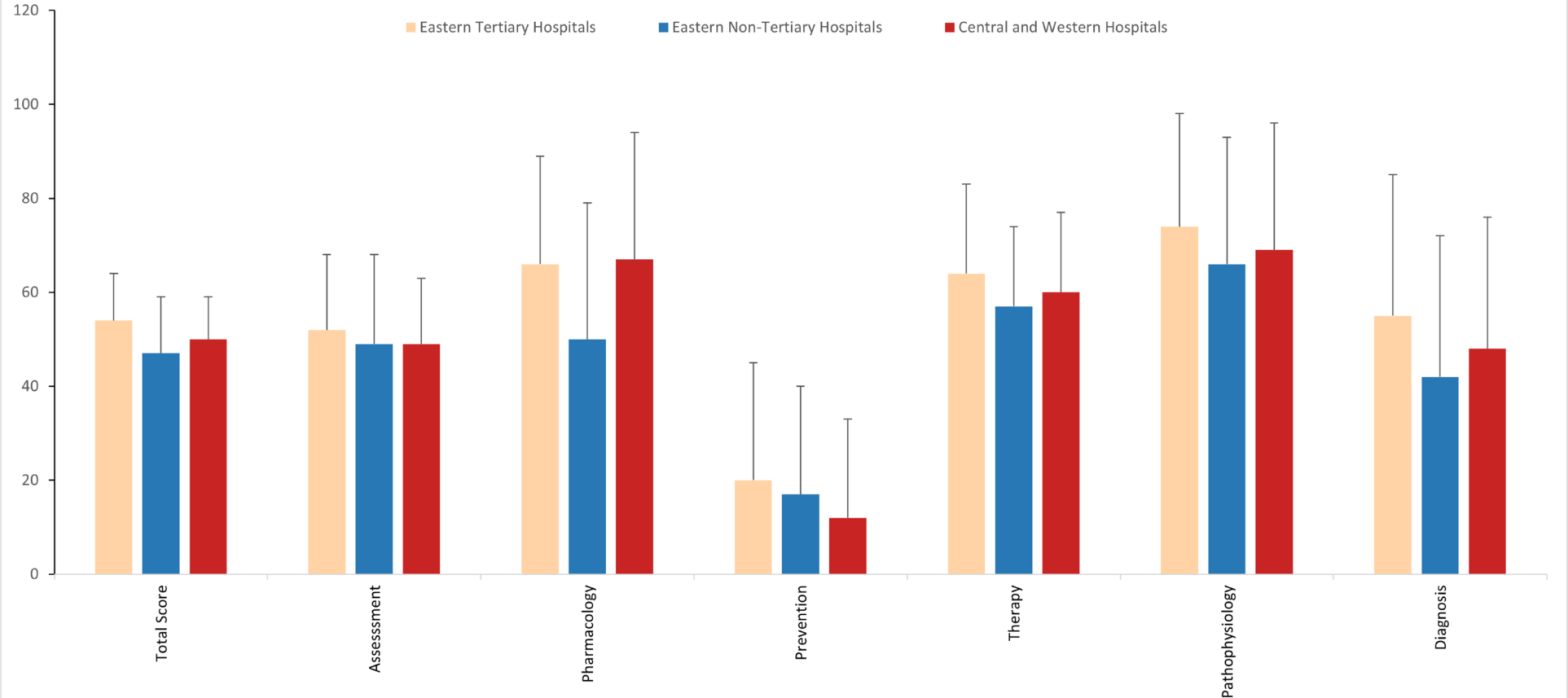
Six core competencies of asthma management in the first round of the test.

Based on the research of the test question proposers, we analyzed the scores of the first round based on six core competencies of asthma management. For the total score, doctors from tertiary hospitals in the eastern region scored the highest, followed by those from hospitals in central and western regions, while doctors from non-tertiary hospitals in the eastern region scored the lowest. Across the six core competencies of asthma management, all doctors scored the lowest in asthma prevention, followed by asthma diagnosis. Doctors from non-tertiary hospitals in the eastern region scored significantly lower in asthma pharmacology compared to doctors from tertiary hospitals in the eastern region and hospitals in central and western regions. In terms of asthma diagnosis, doctors from tertiary hospitals in the eastern region scored significantly higher than those from hospitals in central and western regions and non-tertiary hospitals in the eastern region. No significant statistical differences were observed in the remaining competencies. Figure 1 provides a detailed illustration of these results.

### 3.4 Evaluation of intervention effects

**Figure 2:**
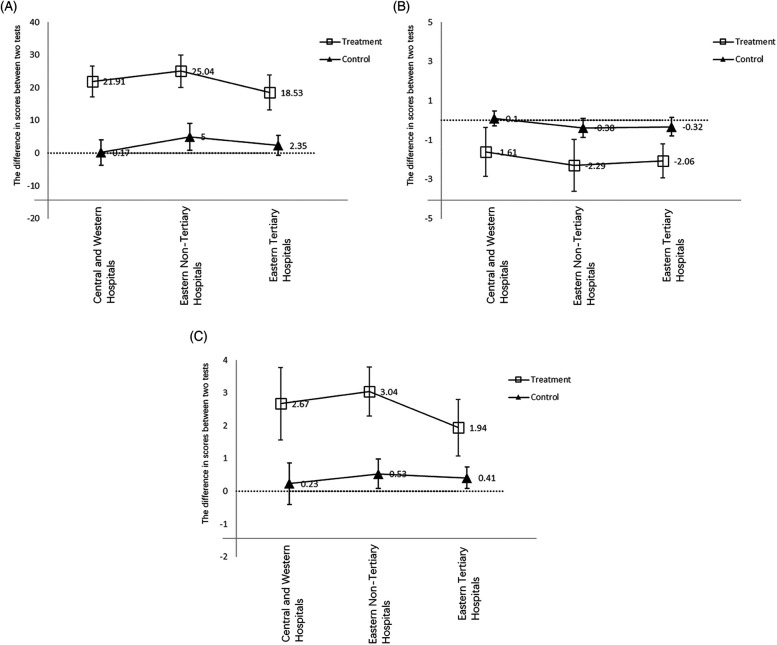
The total scores, difficulty and satisfaction before and after the intervention. **(A)** Scores; **(B)** difficulty; **(C)** satisfaction.

We conducted paired *t*-tests to compare the differences between the intervention group and the control group before and after the intervention. We observed similar trends among participants from different regions, where the intervention group showed significantly higher scores than the control group after the intervention compared to before. Additionally, the intervention improved the satisfaction with answering questions for participants in the intervention group and made answering questions seem easier in the control group, the intervention only increased the scores of doctors from non-tertiary hospitals in the eastern region, and the magnitude of improvement was much smaller than that in the intervention group. Furthermore, the intervention slightly increased the satisfaction with answering questions for doctors from non-tertiary hospitals and tertiary hospitals in the eastern region in the control group, with no significant improvements in other outcome measures. Within the intervention group, the intervention had the greatest impact on the scores and satisfaction with answering questions for doctors from non-tertiary hospitals in the eastern region, followed by those from hospitals in central and western regions, with the smallest impact on doctors from tertiary hospitals in the eastern region. Regarding difficulty, the intervention had the greatest impact on doctors from tertiary hospitals in the eastern region, followed by those from hospitals in the eastern region, and the smallest impact on doctors from hospitals in central and western regions. Figure 2 provides a detailed illustration of these results.

**Figure 3:**
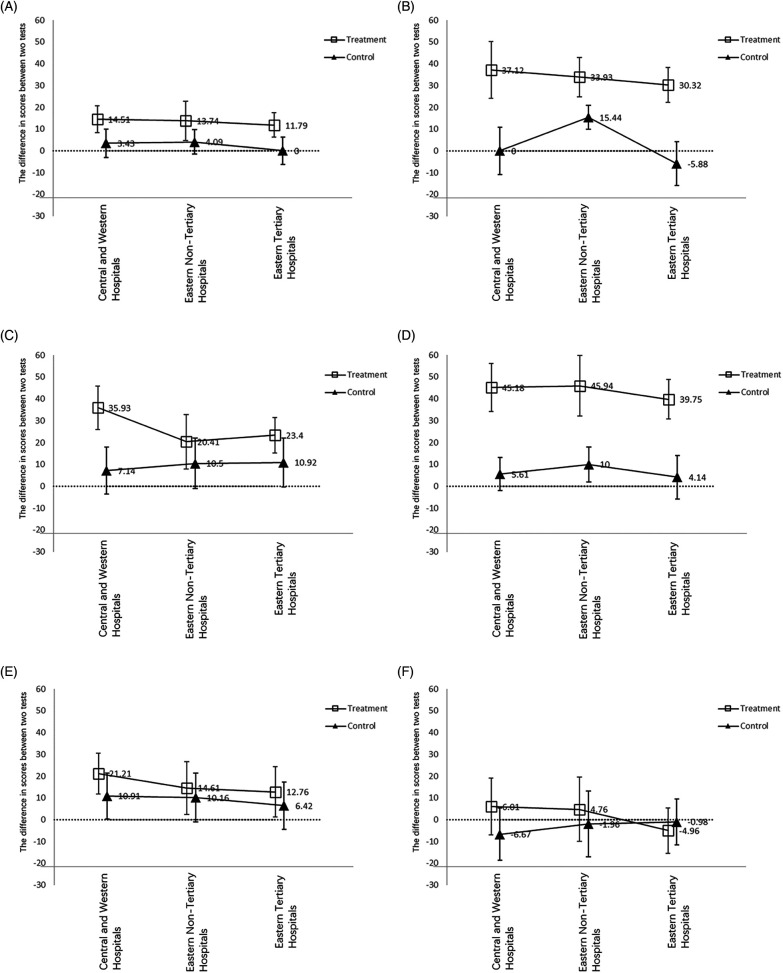
Six core competencies of asthma management before and after the intervention. **(A)** Assessment; **(B)** pathophysiology; **(C)** prevention; **(D)** therapy; **(E)** diagnosis; **(F)** pharmacology.

To further explore the impact of the intervention on the six core competencies, we employed the same method to compare the differences before and after the intervention between the intervention group and the control group. We found that in the intervention group, the intervention did not significantly improve participants' scores in pharmacology. Additionally, the improvement in assessment and diagnosis due to the intervention was lower than the improvement in total scores, while the improvement in pathophysiology and therapy due to the intervention was higher than the improvement in total scores. Figure 3 provides a detailed illustration of these results.

### 3.5 Long-term effects of the intervention

**Figure 4:**
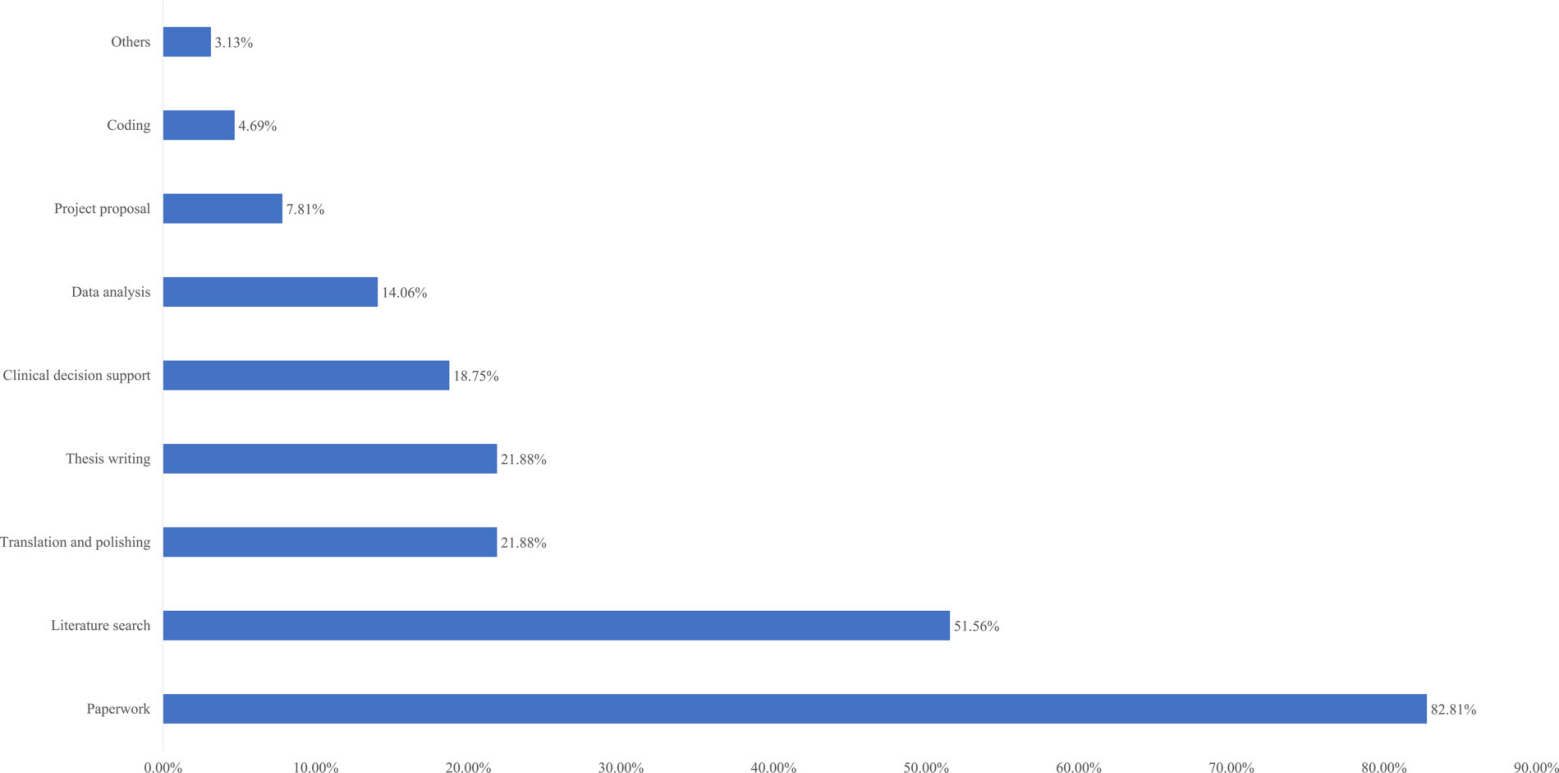
Usage scenarios of ChatGPT in intervention group.

Two weeks after the intervention ended, we conducted an online questionnaire survey of all participants, with a total of 186 questionnaires distributed and 185 returned, resulting in a loss to follow-up rate of 0.5%. We investigated the use of ChatGPT by the control and intervention groups in the past 2 weeks. The usage rate of ChatGPT in the intervention group increased from 6.3% before the intervention to 62%, while the usage rate in the control group remained at a very low level. The intervention group became more familiar with ChatGPT and gave higher ratings for its helpfulness and effectiveness. Table 3 provides detailed information on these results. Additionally, we investigated the usage scenarios of ChatGPT in the intervention group (Figure 4). The top four usage scenarios were paperwork, literature search, translation and polishing and thesis writing.

**Table 3:** Long-term effects of the intervention.

Characteristic	Overall, *N* = 185	Control, *N* = 92	Treated, *N* = 93^a^	*p*-value^b^
Whether to use ChatGPT				<0.001
NO	123 (66%)	88 (96%)	35 (38%)	
YES	63 (34%)	4 (4.3%)	59 (62%)	
Familiarity with ChatGPT	1.95 (3.00)	0.27 (1.35)	3.57 (3.27)	<0.001
Hours spent using ChatGPT per week				<0.001
0	124 (66%)	88 (96%)	36 (38%)	
1–5 h	38 (20%)	3 (3.3%)	35 (37%)	
6–10 h	23 (12%)	1 (1.1%)	22 (23%)	
11–15 h	1 (0.5%)	0 (0%)	1 (1.1%)	
Over 15 h	1 (0.5%)	0 (0%)	1 (1.1%)	
Effectiveness of ChatGPT	2.6 (3.8)	0.3 (1.6)	4.7 (4.0)	<0.001
Satisfaction level with ChatGPT	2.5 (3.7)	0.3 (1.5)	4.7 (4.0)	<0.001
Reliability of ChatGPT	2.43 (3.57)	0.33 (1.58)	4.47 (3.77)	<0.001
Helpfulness of ChatGPT	2.37 (3.46)	0.32 (1.53)	4.36 (3.65)	<0.001
Tendency to use ChatGPT in the future	2.8 (4.1)	0.4 (1.9)	5.2 (4.3)	<0.001
Recommendation level for ChatGPT	2.9 (4.2)	0.4 (1.9)	5.3 (4.3)	<0.001
Having any difficulties while using it				<0.001
Never used	123 (66%)	88 (96%)	35 (38%)	
Had	14 (7.5%)	2 (2.2%)	12 (13%)	
Never had	49 (26%)	2 (2.2%)	47 (49%)	

^a^ *n* (%); Mean (SD).

^b^ Pearson's Chi-squared test; Wilcoxon rank sum test; Fisher's exact test.

## 4 Discussion

According to reports, in rural areas of Beijing, China, the diagnosis rate of asthma is only 48.9%, significantly lower than the 73.9% in urban areas, and only 35.6% of rural asthmatic children receive regular inhaled corticosteroid treatment (9). The improper diagnosis and treatment of asthma in underdeveloped areas are very serious. According to the results of this study, all doctors scored higher in asthma diagnosis than in prevention, ranking second to last. Meanwhile, the differences in scores of participants from different regions in asthma diagnosis were particularly significant, highlighting the urgent need to improve asthma diagnosis skills among doctors in underdeveloped areas and regions on the edge of development.

Furthermore, we found that even with very attractive incentive policies, traditional literature search methods (control group) provided limited assistance in asthma management in the short term. This assistance was significant only in the group of doctors from non-tertiary hospitals in the eastern region, but it only increased by 5 points. We conducted a detailed analysis of the composition of the test questions and found that this improvement was due to an increase in scores related to pathophysiology. In the intervention group, however, we found that using ChatGPT significantly and substantially improved doctors' asthma management abilities in the short term (by approximately 20 points) and increased their satisfaction with answering questions. This effect was particularly pronounced for doctors from hospitals in central and western regions and non-tertiary hospitals in the eastern region.

After using ChatGPT assistance, the average scores of doctors from non-tertiary hospitals in the eastern region and hospitals in central and western regions were 72 ± 8 and 70 ± 12, respectively, surpassing those previously reported for trained asthma specialists (66 ± 6) and asthma specialists (55 ± 6) (20). Furthermore, to explore the role of humans in this process, we conducted 100 rounds of answering the same questions using the same version of ChatGPT (see Supplementary Materials). The results showed that the scores of humans using ChatGPT (70.9) were significantly higher than the scores of ChatGPT responses (64.29). This suggests that the collaboration between humans and ChatGPT achieves a synergistic effect greater than the sum of its parts.

Despite the tremendous assistance that ChatGPT provides to doctors, it is regrettable that, according to our survey, the adoption rate of ChatGPT among Chinese doctors is not high, with only 6.3% having used ChatGPT. In this study, within just 1 h, we managed to increase the adoption rate of ChatGPT among participants from 6.3% to 62%, and many participants indicated their intention to continue using ChatGPT in the future. While we did not stratify post-intervention usage by training level or discipline, future research should explore whether differences in professional background influence the adoption and sustained use of AI tools like ChatGPT. Promoting widespread training on ChatGPT has good feasibility and practical value. Promoting the use of ChatGPT in economically underdeveloped areas is an economically and effective way to address the imbalance in medical development.

This study employed a randomized controlled trial method. It explored the application value of ChatGPT training in addressing the imbalance in regional medical development. It creatively proposed a new method of promoting ChatGPT training in economically underdeveloped areas and regions on the edge of economic development to assist doctors in better patient management. It provided new insights into addressing the imbalance in medical regional development.

To maintain blinding during the assessment process, we adopted simple randomization without stratification. Group allocation was performed before the first round of testing, and participants' asthma-related knowledge was not used as a stratification factor. This design minimized behavioral bias related to group awareness, but may have introduced potential imbalances in baseline knowledge between groups. While the randomization procedure preserved allocation concealment, the absence of stratification by initial knowledge level remains a methodological limitation that should be considered when interpreting between-group differences.

In addition, although the test questions were adapted from a previously published and guideline-based instrument, no formal psychometric validation (such as internal consistency or structural validity) was conducted in our study. This limits the precision with which the test scores reflect physicians’ true knowledge levels. Future studies should consider incorporating standardized validation procedures when using similar instruments.

Due to the limited influence of our medical center, we were unable to include doctors from various regions of China, resulting in a certain bias in the inclusion of the population. Additionally, due to various objective limitations, this study only used one set of test questions to evaluate doctors' asthma management abilities, necessitating a multidimensional evaluation system to increase the credibility of the research. Furthermore, our follow-up only lasted for 2 weeks, requiring longer and more multidimensional follow-ups to evaluate the long-term effectiveness of our intervention. In addition, although the incentive structure in this study was designed to reasonably reflect participants' time commitment and encourage full participation, its potential influence on doctors' willingness to enroll or on their motivation during testing cannot be entirely excluded. While we took measures such as strict eligibility screening and post-enrollment randomization to mitigate such bias, we acknowledge this as an inherent limitation in behavioral intervention studies involving financial rewards.

## Data Availability

The raw data supporting the conclusions of this article will be made available by the authors, without undue reservation.
