# Supplementary material for: Enhancing pediatric asthma management in underdeveloped regions through ChatGPT training for doctors: a randomized controlled trial
Source: Front Pediatr. 2025 Jul 3;13:1519751. doi: 10.3389/fped.2025.1519751 (PMC12267265; doi:10.3389/fped.2025.1519751)
Supplement: Supplementary file 1 [file Image1.pdf]

Supplementary Material

1 Supplementary Figure

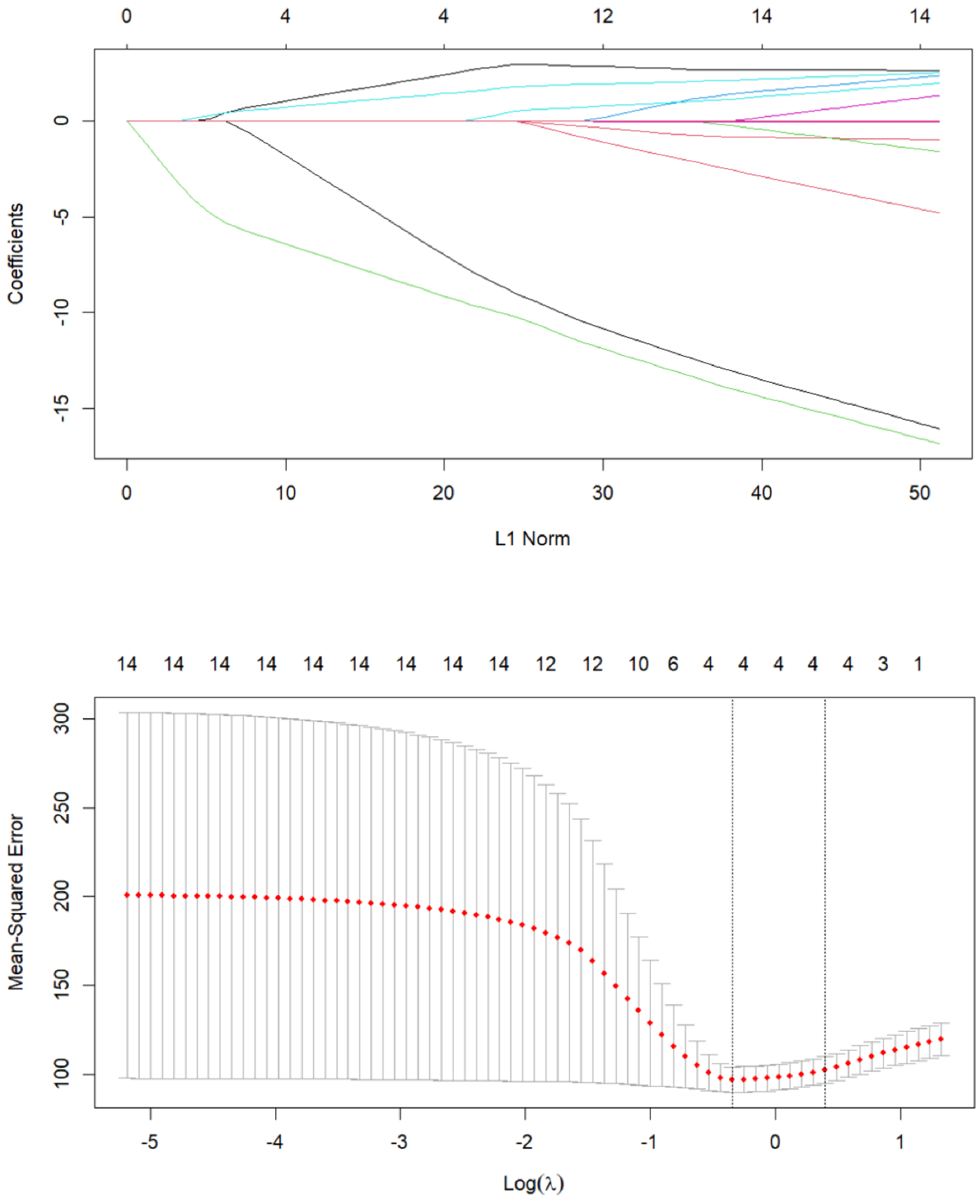

Supplementary Figure 1. The result of LASSO, including Sex, Age, Type of practice hospital, Professional title, Practice Department, Duration of Medical Practice, Weekly Asthma Clinic

Sessions, Number of Asthma Patients Seen Weekly, Weekly Pulmonary Function Tests, Long-term Follow-up Numbers for Asthma Patients, Usage of ChatGPT and group. Paired t-tests were used to examine differences between the first and second rounds of testing.
